# Automated high-throughput image processing as part of the screening platform for personalized oncology

**DOI:** 10.1038/s41598-023-32144-z

**Published:** 2023-03-29

**Authors:** Marcel P. Schilling, Razan El Khaled El Faraj, Joaquín Eduardo Urrutia Gómez, Steffen J. Sonnentag, Fei Wang, Britta Nestler, Véronique Orian-Rousseau, Anna A. Popova, Pavel A. Levkin, Markus Reischl

**Affiliations:** 1 Institute for Automation and Applied Informatics, Karlsruhe Institute of Technology, 76344 Eggenstein-Leopoldshafen, Germany; 2 Institute of Biological and Chemical Systems - Functional Molecular Systems, Karlsruhe Institute of Technology, 76344 Eggenstein-Leopoldshafen, Germany; 3 Institute for Applied Materials, Karlsruhe Institute of Technology, 76131 Karlsruhe, Germany

**Keywords:** Biochemistry, Biological techniques, Biotechnology, Cancer, Cell biology, Chemical biology, Computational biology and bioinformatics, Drug discovery, Diseases, Health care, Medical research, Oncology, Engineering

## Abstract

Cancer is a devastating disease and the second leading cause of death worldwide. However, the development of resistance to current therapies is making cancer treatment more difficult. Combining the multi-omics data of individual tumors with information on their in-vitro Drug Sensitivity and Resistance Test (DSRT) can help to determine the appropriate therapy for each patient. Miniaturized high-throughput technologies, such as the droplet microarray, enable personalized oncology. We are developing a platform that incorporates DSRT profiling workflows from minute amounts of cellular material and reagents. Experimental results often rely on image-based readout techniques, where images are often constructed in grid-like structures with heterogeneous image processing targets. However, manual image analysis is time-consuming, not reproducible, and impossible for high-throughput experiments due to the amount of data generated. Therefore, automated image processing solutions are an essential component of a screening platform for personalized oncology. We present our comprehensive concept that considers assisted image annotation, algorithms for image processing of grid-like high-throughput experiments, and enhanced learning processes. In addition, the concept includes the deployment of processing pipelines. Details of the computation and implementation are presented. In particular, we outline solutions for linking automated image processing for personalized oncology with high-performance computing. Finally, we demonstrate the advantages of our proposal, using image data from heterogeneous practical experiments and challenges.

## Introduction

**Figure 1:**
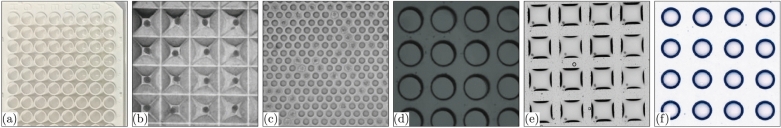
Grid Types. Crops of the grid types (**a**) Microplate^1^, (**b**) Microwell-mesh^2^, (**c**) Agarose Microwells^3^, (**d**) Microwell Array Chips^4^, and (**e**–**f**) DMA^5^ are shown.

Cancer is the second leading cause of death worldwide after cardiovascular diseases. A major problem in cancer therapy is the development of resistance to the current treatment. The combination of in-vitro Drug Sensitivity and Resistance Test (DSRT) and multi-omics data from individual tumors helps determine the appropriate therapy for patients. In modern biotechnology, miniaturized and parallelized systems have become indispensable tools to address high-throughput assays^13,14^. Such systems offer a higher degree of spatio-temporal handling and reduce the workload by up to a thousand-fold while significantly increasing the number of samples per run. This technology improves the performance of the assay. Miniaturized high-throughput technology is typically built with grid structures consisting of hundreds of elements, such as the Microplate^1^, the Microwell-mesh^2^, the Agarose Microwells^3^, the Microwell Array Chips^4^, or the Droplet Microarray (DMA)^5^ (cf. Fig. 1). This technology, for instance, tests the cells for their sensitivity to anti-cancer drugs, which could ultimately be utilized for personalized oncology predictions^15^.

Imaging techniques such as fluorescence microscopy or scanners are often used to visualize and interpret the results of experiments. However, despite the named advantages, these miniaturized high-throughput systems also have limitations. The generated data is substantial and needs to be interpreted. In the project called “Screening Platform for Personalized Oncology” (SPPO), automated image processing is essential, as manual analysis is time-consuming and leads to difficulties in reproducibility.

**Figure 2:**
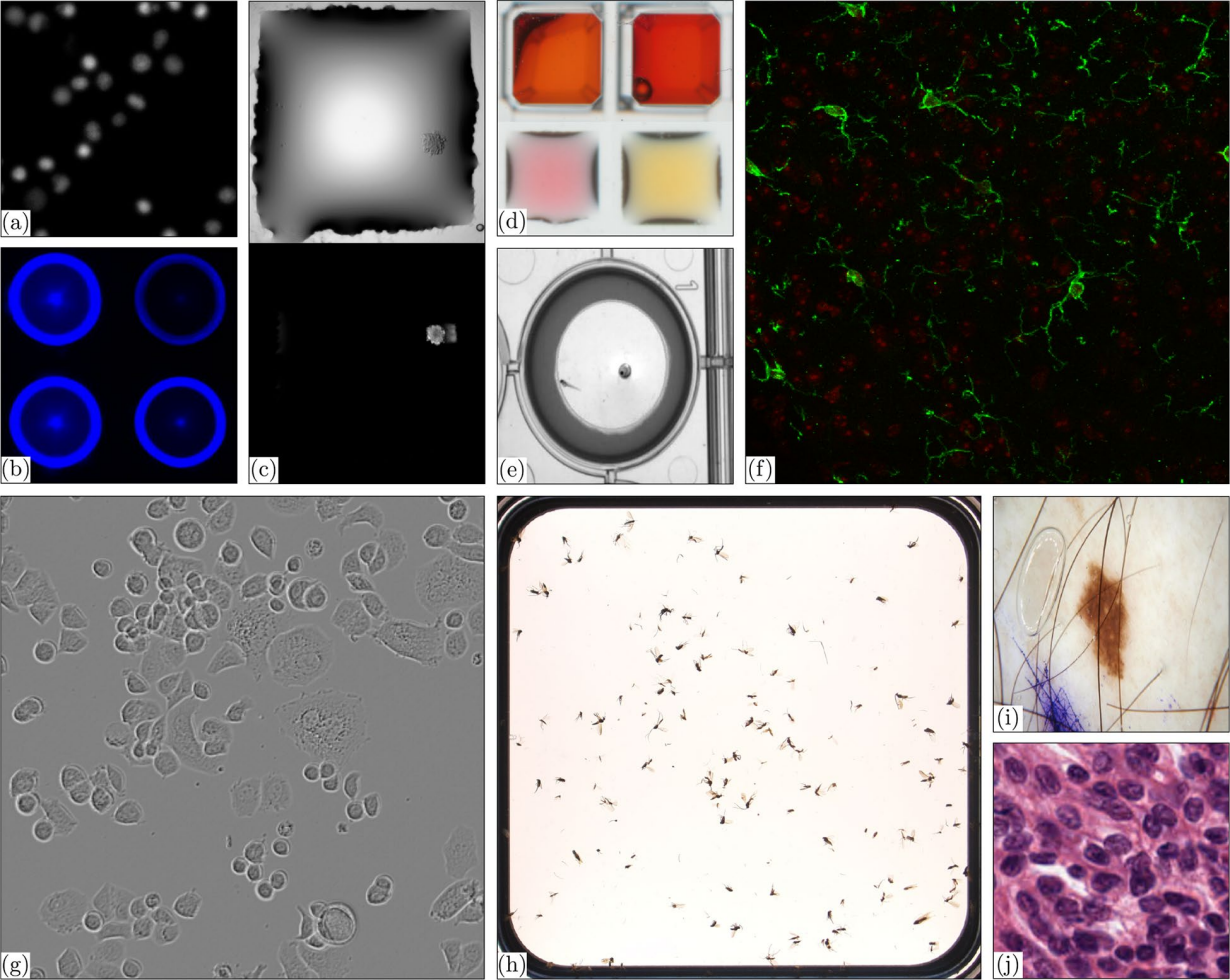
Heterogeneous Image Processing Data and Tasks of High-throughput Experiments. Exemplary tasks, such as (**a**) the detection of cell nuclei in fluorescence images, (**b**) the intensity analysis of spots in fluorescence^6^, (**c**) the detection of spheroids in bright-field or fluorescence images^7^, (**d**) the analysis of colorimetric scans, (**e**) drug screening via touch response analysis of zebrafish larvae^8^, (**f**) the detection of microglia, (**g**) the detection of cells in light microscopy^9^, (**h**) invertebrate biodiversity analysis^10^, (**i**) the inspection of skin lesions^11^, or (**j**) histopathology image analysis^12^, are shown to illustrate the heterogeneity and complexity of high-throughput experiments. To show the generalization of our concept, the presented data is not limited to the SPPO project.

Imaging data and corresponding objectives in the analysis of high-throughput experiments are complex and heterogeneous, as shown in Fig. 2. These characteristics lead to various challenges that make fully automated image data processing complex: (1) Traditional image processing algorithms implemented in common software packages^16,17^ cannot be used for accurate image analysis by default. (2) Domain experts must become familiar with the development/implementation of custom software solutions. (3) In particular, approaches that focus on grid-like structures are not yet available. (iv) High-resolution images combined with many experiments require substantial computational resources.

However, Deep Learning (DL) is suitable for solving heterogeneous problems^18,19^. Nevertheless, several open problems complicate the application of Deep Neural Networks (DNNs). There are many individual DL algorithms, but a comprehensive and extensible concept has yet to be developed. In addition, approaches must be tailored to high-throughput tasks, as given in the SPPO project, which deals with the processing of grid-like structures. An automated grid estimation (macro level) and flexible spot-wise processing (micro level) are required. For instance, the analysis of cell nuclei requires more than a single instance segmentation approach. The calculation of the cell viability necessitates a fusion algorithm or a description of cell features, such as the area or eccentricity.

The scarcity of publicly available annotated datasets for individual high-throughput experiments poses a challenge for the supervised training of DNNs^18^. Currently, there are no comprehensive methods to increase the efficiency and improve the quality of annotations^20^. In addition, an open challenge in the SPPO project is to improve supervised learning processes to reduce the amount of annotated samples required. High-throughput experiments typically demand expertise from multiple disciplines. A workflow is needed that combines the knowledge of the experimenters with the expertise of data scientists. Experimenters often have neither access to GPU hardware nor programming skills. Usability problems, software requirements, or the need for specialized hardware^21^ prevent experimenters from applying automated algorithms.

For interdisciplinary projects, we contribute a comprehensive concept for automated image processing in the context of high-throughput grid data. Methods for assisted image annotation, algorithms for high-throughput image processing, alternative learning processes, and the deployment of pipelines are considered. We also present details on the implementation, computation, and integration of High-Performance Computing (HPC) used to promote the application of our image and data analysis methods. In addition, we show excerpts of results from SPPO and other projects, using heterogeneous image data to demonstrate the generalization ability of our pipeline.

In Sect. “Screening platform for personalized oncology”, we give a brief overview of SPPO. Further, the workflow and methods for automated image processing are described in Sect. “Methods”. Then, an account of computation and implementation is given in Sect. “Computation and implementation. Moreover, Sect. “Results” shows the results obtained, followed by a discussion (cf. Sect. “Discussion”). Finally, we conclude our work in Sect. “Conclusion”.

## Screening platform for personalized oncology

DMA is a miniaturized biocompatible platform based on superhydrophilic superhydrophobic micropatterns on a thin coating layer. A standard glass microscope slide forms the basic structure. The difference in the wettability of hydrophilic and superhydrophobic regions allows the formation of nanodroplets from aqueous solutions that remain stable on hydrophilic spots without physical barriers. This technology has proven its versatility by being tested in various research areas. For example, it has been used as a platform to develop combinatorial organic synthesis^22^ and hydrogel synthesis^23^. Additionally, it has been utilized for advancements in various fields of biology, such as artificial multicellular systems^24^, drug screening^25,26^, embryoid body screening^27^, and DNA delivery^28^. The results of these studies suggest that this arrangement of open nanodroplets offers several advantages over standard methods. These advantages include defined nanoliter-sized compartments, easy and fast access to spots, and low sample/reagent consumption. As a result, the DMA is considered a viable system for personalized medicine. Personalized medicine is one of the main medical directions of our time. However, due to the intrinsic heterogeneity of malignant tumors, the “one size fits all” therapeutic approach is not always efficient. The genetic characteristics of each patient should be taken into account. So-called Patient-Derived Organoids (PDOs) can be established from the primary tumor tissue of the patient, which to a large extent, reflect the tumor heterogeneity in an ex-vivo setting^29^. Studies showed that therapeutic reagents with a positive effect in the context of the PDOs could also be used effectively for the respective patients^29^. Unfortunately, this type of personalized medicine is very time-consuming and expensive. In addition, the DMA platform offers a unique opportunity to combine classical cell biology methods, including cell culture and treatment, with molecular biology techniques, such as proteome and genome extraction protocols, on a single chip (cf. Fig. 1). Another advantage is that a DMA platform is used for cell-based screening^5^. Testing the susceptibility of patient-derived cancer cells to anti-cancer drug treatment in vitro in so-called DSRT is a major clinical development. Using DMA as an SPPO is considered an efficient screening method. Such an SPPO method allows us to evaluate a dose response on each anti-cancer compound per droplet. In addition, this method demonstrates the potential for a highly miniaturized DSRT as a personalized medicine, using only a tiny amount of primary patient-derived cells^15^.

## Methods

### Overall concept

**Figure 3:**
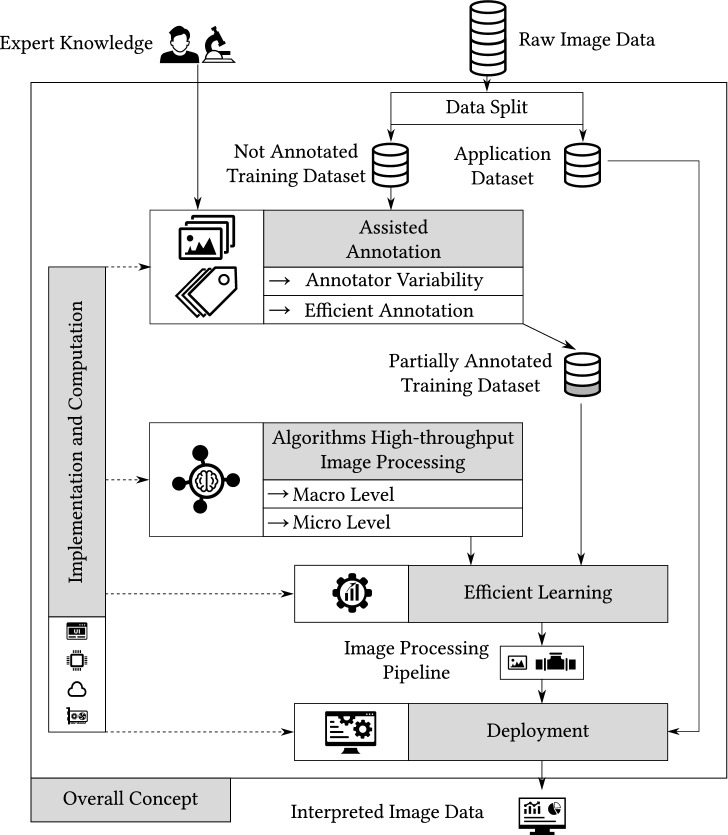
Overall Concept for Information and Data Processing. Raw image data is split into an application dataset and not annotated training dataset. Considering efficiency and annotator variability, a partially annotated training dataset is then created using expert knowledge. The partially annotated dataset is the starting point for parameterizing the developed algorithms for high-throughput image processing, considering both the macro and the micro level. The final image processing pipeline emerges after the learning process is complete. Then, in the application deployment step, the pipeline can be rolled out to generate interpreted image data for the remaining application dataset or emerging data. In general, solutions related to implementation and computation are required in all concept elements.

The proposed concept for automated image processing within SPPO is illustrated in Fig. 3. Raw image data and expert knowledge form the starting point of the image processing problem. First, the data is divided into an application and not annotated training dataset, which is relevant for further investigations into machine learning. Then, the assisted annotation, which focuses on annotator variability and increasing efficiency, forms the basis for generating a partially annotated dataset from expert knowledge. A detailed presentation of this can be found in Sect. “Assisted annotation”.

Furthermore, tailor-made image processing algorithms for the high-throughput application form another concept component, which is explained in Sect. “Automated high-throughput image processing”. Both the macro and micro levels are addressed in the context of image processing. The macro level deals with the analysis of the grid, while the micro level considers the individual elements of the grid.

Another part of the concept investigates elaborated learning methods compared to state-of-the-art supervised learning approaches. The focus is on parameterizing robust image processing pipelines with as little annotated data as possible.

Finally, the concept deals with deploying algorithms to make them useful for researchers in practice. Here, we contribute to bridge the gap between the development and usage of image processing pipelines. This element relates to the implementation and computation component (cf. Sect. “Computation and implementation”), which focuses on implementing all methods in real-world applications.

### Assisted annotation

**Figure 4:**
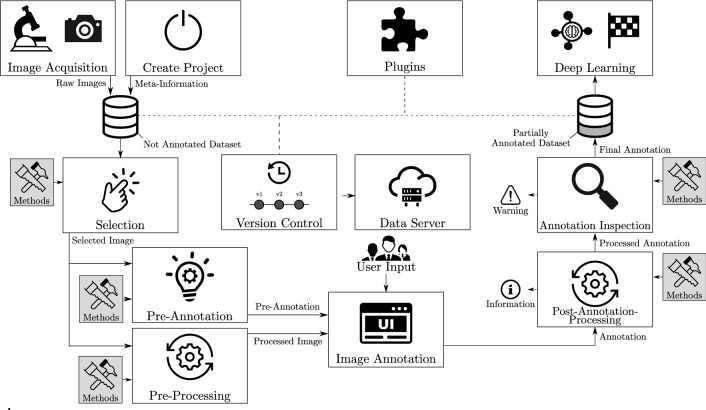
Assisted Annotation. The workflow of the proposed assisted annotation approach is visualized^20^. Via “Selection”, the most informative samples are obtained. “Pre-Processing” aims to make annotation easier for users. “Pre-Annotation” provides an initial annotation that a user adapts during “Image Annotation”. The remaining errors are managed by “Post-Annotation-Processing” (integrating prior knowledge to optimize annotation quality automatically) and “Annotation Inspection” (triggering warnings in case of a violated quality criterion).

Figure 4 presents an overview w.r.t. the proposed method for assisted annotation, which is based on the publications of Schilling et al.^20,30,31^.

A raw image dataset obtained during data acquisition is supplied with meta-information that is essential for scientific data management. Inspired by deep active learning, the component “Selection” allows users to influence the order of images presented to them. The goal is to focus on the most promising images. When selecting samples, for instance, methods consider the heterogeneity of samples or the uncertainty of an already available DNN. There are various scenarios where the annotation of raw images is a challenge. The pre-processing element addresses this issue. It enables image pre-processing inherently, i.e., by normalizing or cropping images to simplify the annotation for users. The concept of pre-annotation incorporates available algorithms (i.e., pre-trained DNNs or traditional image processing algorithms such as Otsu) that can serve as heuristics and provide an initial estimate. Therefore, users only need to correct the predicted annotations. Adapting the input interface to the image processing tasks guarantees task independence. The post-processing of annotations directly avoids recurring errors by incorporating prior knowledge, e.g., that there are no holes in segments.

Besides, the annotator variability problem is considered (cf. annotation inspection). Quality criteria, like a comparison of the annotation and prediction of an inspection DNN, can be evaluated to trigger warnings on suspected errors. Since the form of support is project-dependent, extensions or customizations can be integrated due to the generic design. In addition, we integrate dataset version control capability to track the dataset history and simplify the transfer to data servers. Finally, plugins consider neighboring image annotation activities and integrate elements, such as DNN training, the application of trained DL pipelines, or the post-processing of predictions by DNNs.

### Automated high-throughput image processing

**Figure 5:**
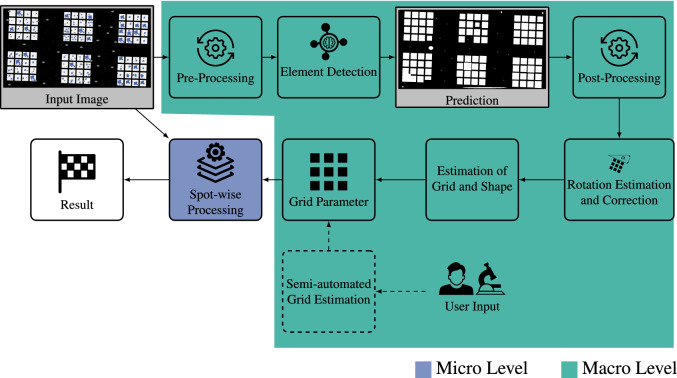
Automated High-throughput Image Processing. The grid parameters are determined at the macro level () by pre-processing an input image to enhance the subsequent element detection based on DL, using the post-processed predicted segments to estimate/correct the grid rotation (cf. Sect. “Automated high‑throughput image processing”). Besides, semi-automated grid estimation (dashed) is possible. The macro level is followed by spotwise-processing (cf. Sect. “Automated high‑throughput image processing”) at the micro level ()^32^.

Figure 5 shows the developed approach for automated high-throughput image processing based on the work of Schilling et al.^32^. The method is composed of two components: (i) automated grid parameter estimation (macro level, cf. Sect. “Automated high‑throughput image processing”) and (ii) spot-wise processing (micro level, cf. Sect. “Automated high‑throughput image processing”), which are discussed below.

#### Grid detection

First, the input image is pre-processed, i.e., conversion to grayscale, allowing the processing of different image types and normalization to improve the performance of the subsequent DNN for spot detection. Next, a DNN is used for segmentation to distinguish between pixels belonging to elements or the background. Various post-processing operations, such as smoothing with morphological operators or filtering based on the area of an element, further optimize the robustness of the detected elements. A sophisticated algorithm that considers the arrangement of neighborhood elements is used to estimate and correct the rotation of the grid. Subsequently, the grid parameters can be obtained by using robust parameter estimation approaches. In addition, a semi-automated grid estimation enables the estimation of grid parameters in cases where the automated approach fails, i.e., challenging imaging conditions. However, this method requires user input as a subset of grid parameters.

#### Selected methods for spot-wise processing

**Automated object detection** Object detection is a common problem in biomedical applications. For example, counting the number of cells or describing the properties of organoids are of particular interest.

**Figure 6:**
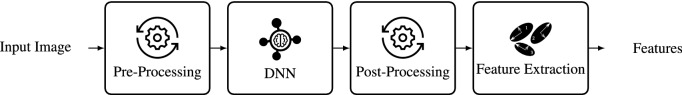
Automated Object Detection Pipeline. The input image is pre-processed before being fed into a DNN. Then, the DNN prediction is post-processed to obtain objects described by different properties using feature extraction.

Figure 6 presents our proposed pipeline for automated object detection inspired by Scherr et al.^33^. First, the input image is pre-processed by normalization to obtain a reasonable range of values for the subsequent DNN. In the case of instance segmentation, the DNN predicts Euclidean distance maps that are post-processed using a seed-based watershed algorithm to obtain objects. In semantic segmentation, the prediction of the DNN is post-processed by selecting the class with the highest output value. A feature extraction step follows to export various object properties, such as the area, the position of the centroid, or the length of the major/minor axis.

**Figure 7:**
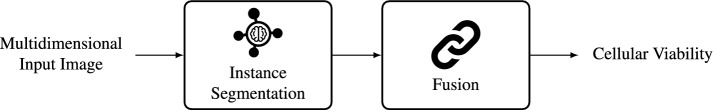
Automated Cellular Viability Analysis Pipeline. A multidimensional input image is processed with the presented approach of instance segmentation. A fusion step is required to obtain cellular viability.

**Automated cellular viability analysis** Ex-vivo drug screening assays test the cellular viability after drug treatment at appropriate dilutions. The proposed novel image processing pipeline for cell viability analysis is shown in Fig. 7. The multidimensional input image is processed channel by channel using the instance segmentation approach presented (cf. Sect. “Automated high‑throughput image processing”). The Hoechst channel stains the nucleus of all cells, dead or alive, giving the total number of cells. Calcein indicates only living cells, and PI stains the nucleus of cells containing ruptured cell membrane (dead cells). However, some cells appear to stain faintly for Calcein, while at the same time being already positive for PI.

In a fusion step, the information is merged. Using the k-nearest-neighbor algorithm, matching instances between the different staining channels are obtained. Then, cellular viability can be calculated by comparing the total number of cells with the instances that meet the specified criteria (Hoechst/Calcein positive, but PI negative).

**Colorimetric analysis** In many high-throughput experiments, colorimetric analysis of scanner images can be used to conclude experimental results. The objective of image processing is to automatically calculate a quantitative metric per spot element that correlates with the output variable of interest. The metrics can thus be converted into each other via a calibration measurement. Next, an abstract transformation function is used to convert the RGB value into a scalar, which is utilized to quantify the color of the element. The transformation functions depend strongly on the particular experiment and the given color spectrum. Examples are color space transformations or individual linear combinations of the RGB values.

### Deployment

There are special requirements within the interdisciplinary SPPO project. The automated image processing pipeline should be usable by experimenters to avoid cumbersome workflows and provide direct image analysis without waiting time. Therefore, a user-friendly framework is needed that does not require special hardware, is out-of-the-box, and does not require complicated software installation procedures or dependencies on commercial solutions.

Our deployment framework proposes to provide cross-platform, open-source software packages, including Graphical User Interfaces (GUIs) and user manuals. The deployment of the software packages consists of four parts: 1) A local installation on a user device is possible. 2) We propose the establishment of an image processing server equipped with the required software/hardware that can be accessed via a remote desktop connection, independent of the local device. This solution is designed for basic computations such as assisted image annotation. 3) We provide a RestAPI solution that connects the image processing server to a single Graphics Processing Unit (GPU) for medium-scale computations like inference steps of newly collected experimental data.

4) An integrated GUI-based submission system that interfaces with the HPC workload manager SLURM allows experimenters to use HPC resources without coding. Having access to an HPC cluster brings several advantages. For instance, training a DNN with a single GPU instead of a CPU or with multiple GPUs by applying data-parallel training approaches are use cases of HPC that shorten the training duration^34^. In addition, the cluster enables high-throughput computing because multiple computational nodes are equipped with several GPUs. Taking parallel independent hyperparameter optimizations or DNN inference into account, the benefit from the perspective of high-throughput computing emerges. The computational benefit scales approximately linearly with the number of devices available^34^.

In addition, our solutions consider a global data server for seamless data exchange between all computing systems. For further details, refer to the publications^20,32,34^.

## Computation and implementation

**Figure 8:**
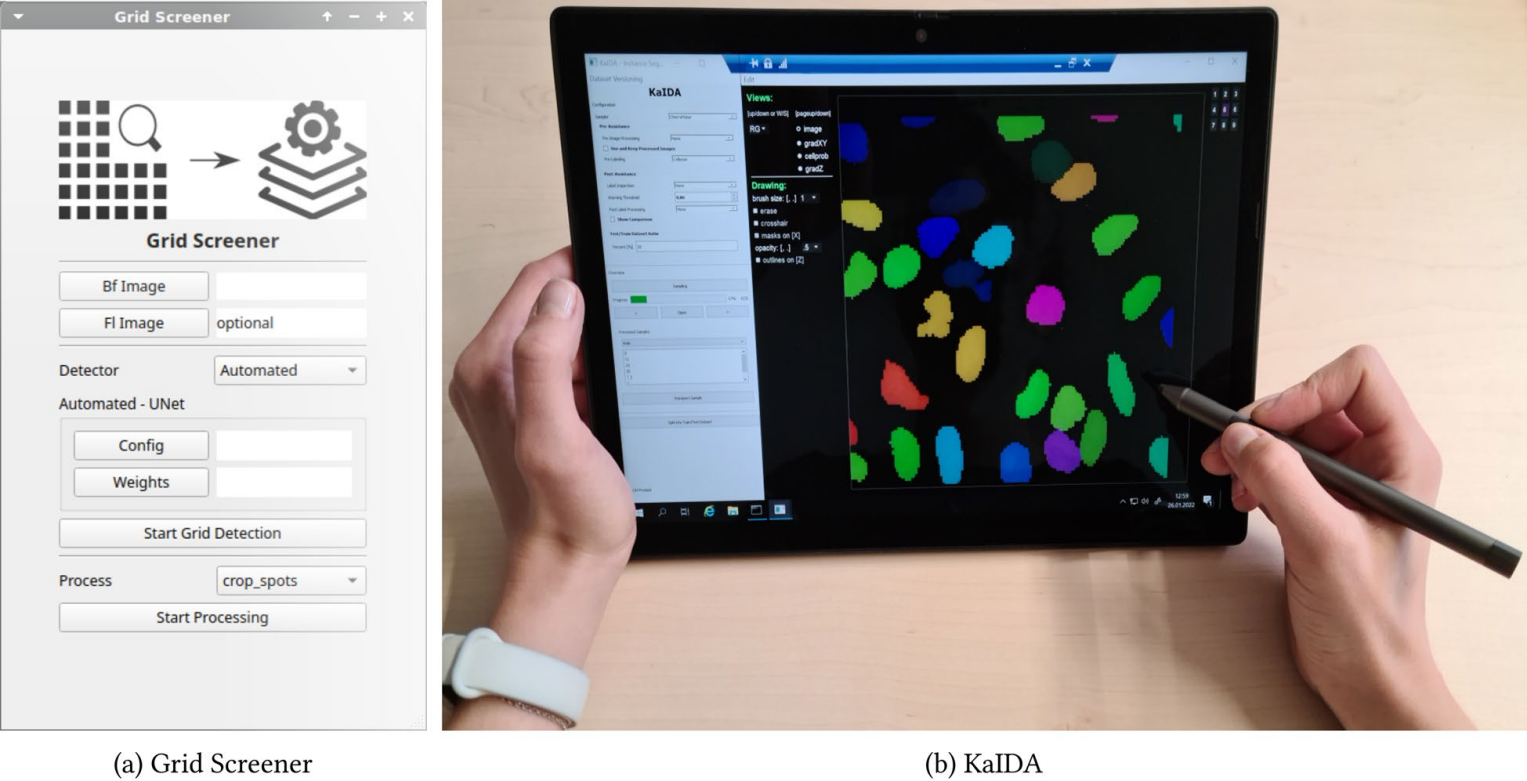
Overview GUIs. The GUI of Grid Screener^32^ (**a**) is shown. Besides, the usage of KaIDA^20^, integrated into a Lenovo x12 Detachable mobile touchscreen device (**b**) is visualized.

We have developed an extensive open-source software portfolio with GUIs available as cross-platform Python pip packages. The tool Grid Screener^32^, available at https://git.scc.kit.edu/sc1357/grid-screener, covers the implementation of the proposed methods for automated high-throughput screening. The Karlsruhe Image Annotation Tool (KaIDA)^20^ software package implements our proposal for assisted image annotation. The repository is located at https://git.scc.kit.edu/sc1357/kaida. The GUIs can be found in Fig. 8.

We consider the Large Scale Data Facility (LSDF)^35^ as a global data server system. In addition, we set up a prototype server described in Table 1. Moreover, we provide researchers with a Lenovo x12 Detachable device that can be connected to the image processing server. This hardware offers a touchscreen for image annotation (cf. Fig. 8). The RestAPI solution is connected to a local GPU server (GPU: Nvidia Tesla V100, CPU: Intel Xeon 5118 CPU).

**Table 1:** Prototype Server. Details w.r.t. established prototype server^20^ are shown.

Elements	Description
Processing Server	Operating System: Windows Server 2019
CPU	Intel Xeon 4210R
GPU	Nvidia Quadro RTX 4000 (8 GB)
Local Storage	RAM: 32 GB, 512 GB SSD
Update	Git Version Control

Furthermore, the SLURM submission system integrated into KaIDA is linked to HoreKa, which allows elaborate DNN training or inference on HPC resources. A computational node of HoreKa is equipped with an Intel Xeon Platinum 8368 CPU (2 sockets, 76 cores per socket) and four NVIDIA A100 Tensor Core GPUs. This configuration enables high-performance and high-throughput computing. There are a total of 167 nodes available.

## Results

We give an overview of our investigations on automated high-throughput image processing within the SPPO project and other projects to demonstrate the generalization ability of our concept. However, presenting the results of all modules given in our concept would go beyond the scope of this paper. Therefore, only excerpts from the results are shown below. Please refer to^20,30–32,34,36^ for further details.

### Assisted annotation

In the following, the results of the modules “Selection” and “Pre-Annotation” are presented in detail.

#### Selection

**Figure 9:**
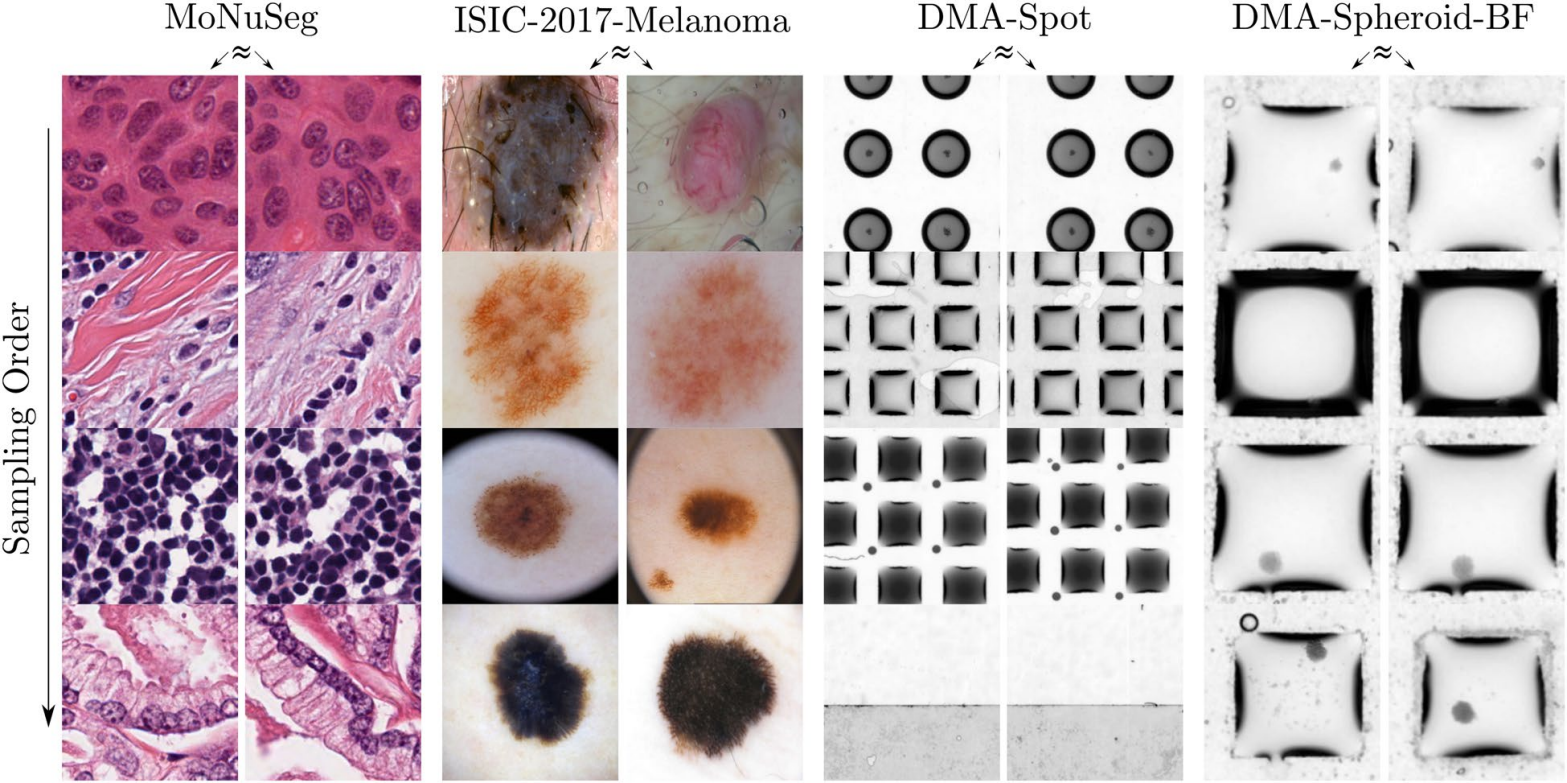
Automated Selection Based on Dataset Heterogeneity. Using ResNet18 in combination with PCA on ten features, the selection order (left row) for different datasets (MoNuSeg^12^, ISIC-2017-Melanoma^11^, DMA-Spot, DMA-Spheroid-BF) is visualized. In addition, the images with the highest similarity ($\approx$) are compared row by row.

The results of the selection of samples based on dataset heterogeneity are shown in Fig. 9^37^. This experiment combines an ImageNet pre-trained ResNet18^38^ with a principal component analysis. The purpose of this combination was to generate a ten-dimensional feature space that can be used to automatically select heterogeneous images of a dataset that should be annotated. A visual inspection of the selection order reveals that the selected images (vertical direction) are dissimilar. Furthermore, the nearest neighbors in the feature space have substantial visual similarities (horizontal direction). Hence, the “Selection” module can provide annotators with different samples to reduce the annotation overhead.

#### Pre-annotation

**Figure 10:**
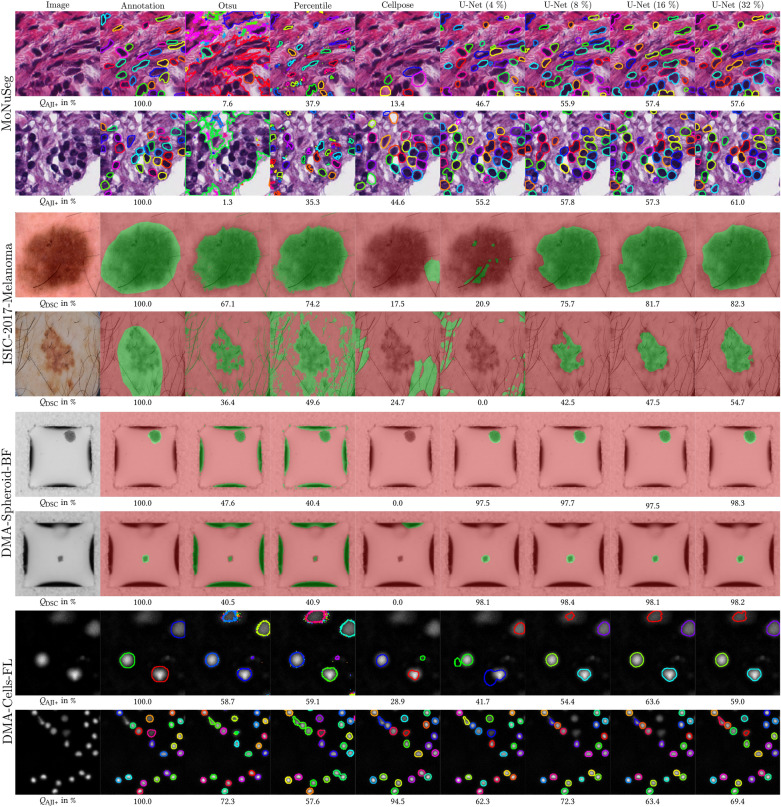
Pre-Annotation. The results of different pre-annotation methods (Otsu, percentile threshold, Cellpose^39^, U-Net^40^) are shown. Example images (first column) of the data introduced in Fig. 2 are illustrated compared to the annotation (second column). Only the U-Net has been trained on an already annotated sub-dataset. Here, 4% to 32% of all images in the dataset were annotated in advance. The other methods do not require an annotated sub-dataset. In addition to qualitative results in the form of contours or overlays, quantitative metrics (Dice Coefficient $Q_{\text {DSC}}$ or advanced Aggregated Jaccard Index $Q_{\text {AJI+}}$) are presented.

Furthermore, the results of different pre-annotation methods (Otsu, percentile threshold, Cellpose^39^, U-Net^40^) will be discussed, which are evaluated w.r.t. the introduced data in Fig. 2. The results are displayed in Fig. 10, where two examples per dataset, including the original annotation, are shown compared to different pre-annotation methods. In addition, the performance of methods per image is qualitatively shown using overlay/contour and quantitatively by metrics (Dice Coefficient $Q_{\text {DSC}}$/ advanced Aggregated Jaccard Index $Q_{\text {AJI+}}$). The core message of the evaluation is that the suitability of the particular method for pre-annotation depends on the problem/dataset. Therefore, as suggested in the concept, it is helpful to be able to select different methods based on the problem. In this context, a DNN (here: U-Net^40^) trained on a small dataset is a generic method. However, the method has the disadvantage of requiring an already annotated dataset for training.

Moreover, the trend shows that the prediction accuracy improves when a more extensive set of annotations is available for the initial training of the DNN. However, the results do not show a monotonic progression of the learning curve, i.e., the performance as a function of the annotation rate, for all datasets. To sum up, the amount of correct segmentation in the pre-annotation step is often superior to the remaining errors and, therefore, advantageous.

### Automated high-throughput image processing

#### Grid detection

**Figure 11:**
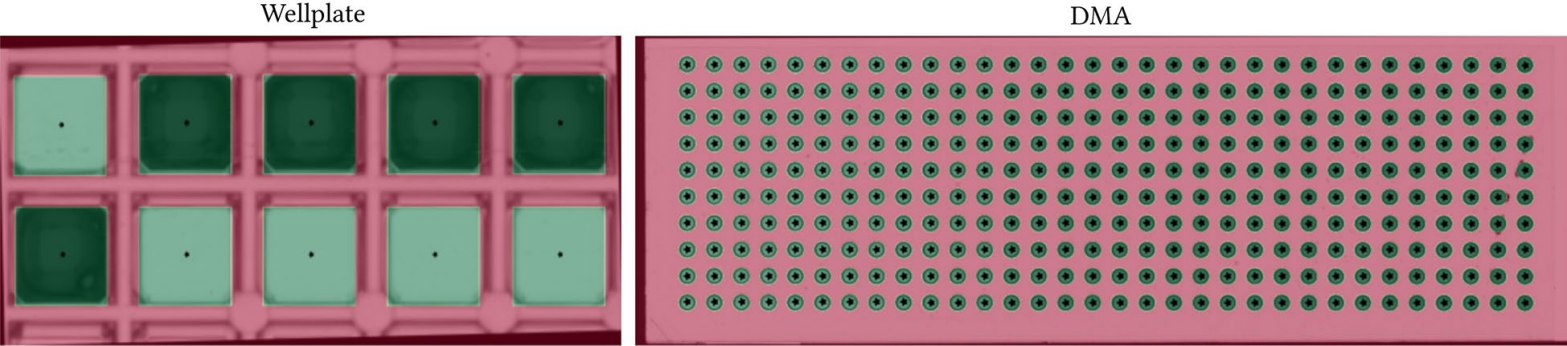
Grid Detection. The automated grid estimation using Grid Screener is shown for wellplate and DMA input images. The grid elements are marked via crosses (centroid) and green overlays. A red overlay denotes the background.

The result of automated grid estimation (DMA slide, wellplate) using Grid Screener is given in Fig. 11. Taking the quantitative results depicted in the work of Schilling et al.^32^ into account, relative errors concerning the estimated geometric grid properties are in the range of 2–3 %. It demonstrates that all grid elements can be detected correctly. Further, various grid formats are manageable, as no difference in accuracy can be observed when comparing different grid structures^32^. Automated processing is more than 200 times faster than manual grid analysis^32^.

#### Spot-wise processing

**Automated object detection** Figure 10 has already demonstrated the superiority of DL-based approaches (U-Net, Cellpose) compared to traditional algorithms (Otsu, percentile threshold). In addition, Table 2 presents an exemplary feature list for an element. The features depend on the respective application. Here, the features area, eccentricity, mean gray value, and the lengths of the major axis/minor axes per instance are shown, which are generated and stored fully automatically. Moreover, the Supplementary material contains further results.

**Table 2:** List of features. The exemplary features (area, eccentricity, mean gray value, major axis/minor axis lengths) extracted per instance are shown.

	ID	Area	Eccentricity	Mean intensity	Major axis	Minor axis, ...
	1	153	0.835	136.784	19.394	10.683
	2	320	0.238	110.491	20.482	19.893
	3	304	0.304	121.214	20.16	19.206
	4	296	0.347	113.936	20.054	18.81
	5	246	0.399	140.801	18.496	16.962
	6	169	0.902	104.538	22.767	9.835
	7	363	0.38	83.59	22.412	20.735
	8	305	0.476	121.069	21.018	18.486

**Figure 12:**
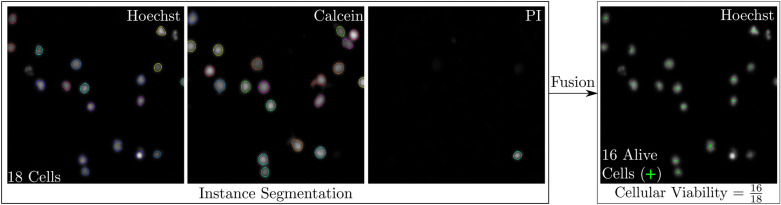
Automated Cellular Viability Analysis. The detected instances in the different stainings (Hoechst, Calcein, PI) are visualized by marking the boundaries. Thereby, different instances are encoded by changing colors. At the end of the fusion step, live cells (Hoechst positive, Calcein positive, PI negative) needed to obtain cellular viability are marked with a green cross. For reasons of visualization and clarity, the images shown represent only a crop of an entire DMA spot.

**Automated cellular viability analysis** An exemplary result of the automated cellular viability analysis using stained fluorescence images of patient-derived CLL cells is given in Fig. 12. The detected instances in the different staining (Hoechst, Calcein, PI) are visualized by marking the cell boundary. Different colors encode different instances. At the end of the fusion step, truly alive cells (Hoechst positive, Calcein positive, PI negative; cf. green crosses) are obtained to calculate cellular viability. This procedure is necessary because some cells in the dying stage are difficult to detect. After overlaying the images with the three channels (Hoechst, Calcein AM, and PI), one cell can simultaneously be Calcein and PI positive. The membrane of the cell is ruptured, but the activity of the protein is still present and metabolizing, indicated by Calcein positive. Another observed aspect is that some only Hoechst positive cells are neither Calcein positive nor PI. This finding explains that the cellular membrane is intact since it is PI negative, but inside, cellular proteins are not active and metabolizing (not indicated by Calcein fluorescent dye). Therefore, detecting the valid number of alive cells and avoiding false positive detection is necessary. This objective is accomplished with our pipeline to overlay all three channel images and calculate the number of alive cells. For reasons of visualization and clarity, the images shown represent only a crop of an entire DMA spot. Thanks to the developed automatic image processing pipeline, cellular viability can be calculated in high-throughput experiments. No manual user interaction is required. Instead, with the help of a DNN, accurate results are obtained according to the defined objectives in assisted image annotation.

**Figure 13:**
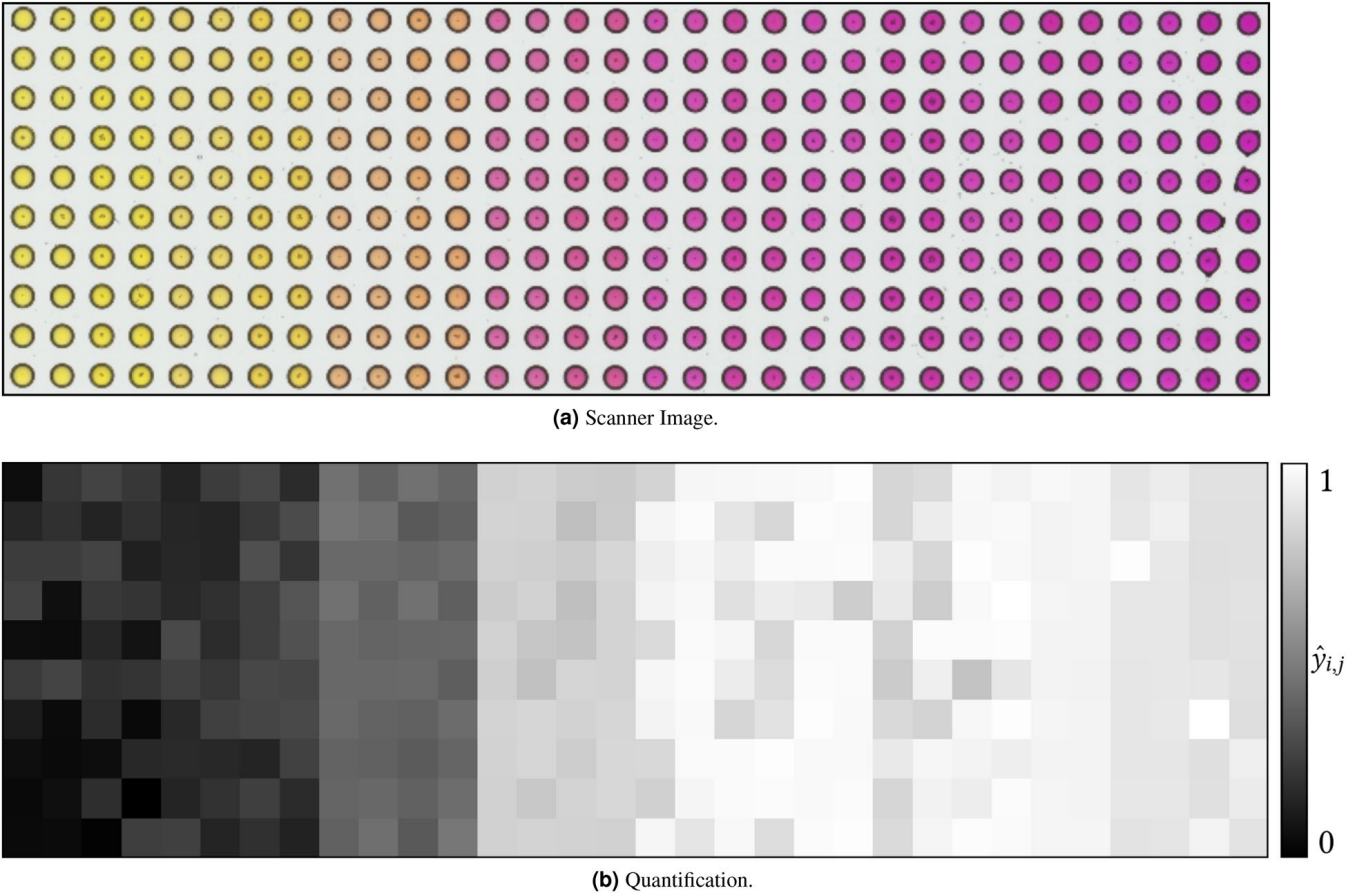
Colorimetric Analysis. An exemplary scanner image (**a**) and the corresponding quantification $\hat{y}_{i,j}$ (**b**) are depicted.

**Colorimetric analysis** Figure 13 displays an exemplary scanner image (a) and the resulting quantification (b) in the context of colorimetric analysis. Since the true values are known (pH values) from a calibration measurement, the correlation between metric $\hat{y}_{i,j}$ and true value can be calculated to evaluate the results. A correlation coefficient of $\rho =0.894$ coincides with the visual evaluation by comparing Fig. 13a,b. Hence, we demonstrate that our concept enables automated colorimetric analysis. In addition, we present more results in the supplementary material.

### Deployment

We want to highlight the advantages of our deployment concept for a successful application. First, although the participants in our project use three different operating systems and different devices, there are no problems regarding the installation/use of the software due to the image processing server. None of the experts has a GPU available locally, but everyone can access it remotely with our concept.

The benefits of our deployment concept will be illustrated by the data characteristics of a DMA experiment (672 spots) to determine cell viability. A single experiment consists of four images (a bright field channel to capture the grid elements and three fluorescence channels). The resolution of the data is 4 $\times$ 12800 px $\times$ 37888 px (16-bit), resulting in a size of 3.7 Giga Bytes per single experiment. Therefore, processing the data with a consumer CPU is impractical, and consumer GPUs also reach their memory limits. In addition, the necessity of a data management system becomes clear since transferring the data by e-mail or similar means is impossible. This example illustrates the demand for the concept we have developed for the SPPO project.

## Discussion

We can detect various grids with the Grid Screener and reduce the effort for experimenters when analyzing high-throughput assays. Further, the examples presented illustrate the heterogeneity in biological and chemical grid-like high-throughput experiments, such as DMA. The results demonstrate the potential of DL tailored to high-throughout screening - its generic applicability and accuracy. A typical experiment may consist of tens of thousands of images, highlighting the advantage of a fully automated processing approach. Therefore, integrating DNNs as methods for automated image analysis is essential for the success of the SPPO project. Manual activities during the evaluation of experiments can be drastically reduced. The remaining bottleneck of image annotation, when using DL, is solved by the proposed assisted annotation. Our concept considers the whole procedure, from raw data to the application of a developed image processing pipeline (deployment, implementation, and computation). Furthermore, this approach simplifies workflows and provides experimenters with the benefits of DL. The computational benefits of accessing HPC clusters are demonstrated, i.e., using powerful computing resources without requiring local hardware.

However, a limitation of our concept is that any computational resource (HPC cluster, local GPU, or GPU server) is required. Further, if no processing server is available, our software must be installed by users. A browser-based web interface or executable is currently not available.

## Conclusion

With our proposed concept, we contribute to the research community by developing a workflow for automated image processing of grid-structured high-throughput experiments in interdisciplinary projects. We provide methods for assisted image annotation and present our tailored algorithms for high-throughput image processing. Finally, we show a strategy for deploying developed processing pipelines. In particular, we consider implementation and computation in our concept to ensure applicability. Several heterogeneous exemplary results have been used to illustrate the advantages of our proposal. In addition, the proposal could be a starting point for other interdisciplinary data analysis projects, as the basic ideas are larger than the SPPO project. We also highlight the benefits of integrating HPC clusters into our project, i.e., the reduction of the DNN training duration or a faster experiment analysis.

Current research investigates annotation-efficient learning processes to reduce the amount of annotated images needed. Furthermore, due to the data heterogeneity in the SPPO project, developing additional spot-wise image processing pipelines tailored to new experiment types is an ongoing work package. Besides, the creation of a web interface/executable of our software tools is being explored. Moreover, an exemplary end-to-end study that shows all elements of our concept in detail is in preparation.

## Supplementary Information

Supplementary Information.

## Data Availability

The DMA datasets and Insects2022 dataset analyzed during the current study are available from the corresponding author upon reasonable request. MoNuSeg is available at https://drive.google.com/file/d/1ZgqFJomqQGNnsx7w7QBzQQMVA16lbVCA/view?usp=sharing, ISIC-2017-Derma at https://challenge.isic-archive.com/data/, and LIVECell at https://sartorius-research.github.io/LIVECell/. The tools are accessible at https://git.scc.kit.edu/sc1357/grid-screener and https://git.scc.kit.edu/sc1357/kaida.
