# Supplementary material for: Automated high-throughput image processing as part of the screening platform for personalized oncology
Source: Sci Rep. 2023 Mar 29;13:5107. doi: 10.1038/s41598-023-32144-z (PMC10060403; doi:10.1038/s41598-023-32144-z)
Supplement: Supplementary file 1 — Supplementary Information. [file 41598_2023_32144_MOESM1_ESM.pdf]

Automated High-throughput Image Processing as Part of the Screening Platform for Personalized Oncology

Marcel P. Schilling[1,*], Razan El Khaled El Faraj[2], Joaquín Eduardo Urrutia Gómez[2], Steffen J. Sonnentag[2], Fei Wang[3], Britta Nestler[3], Véronique Orian-Rousseau[2], Anna A. Popova[2], Pavel A. Levkin[2], and Markus Reischl[1]

[1]Institute for Automation and Applied Informatics, Karlsruhe Institute of Technology, 76344 Eggenstein-Leopoldshafen, Germany
[2]Institute of Biological and Chemical Systems - Functional Molecular Systems, Karlsruhe Institute of Technology, 76344 Eggenstein-Leopoldshafen, Germany
[3]Institute for Applied Materials, Karlsruhe Institute of Technology, 76131 Karlsruhe, Germany
[*]marcel.schilling@kit.edu

SUPPLEMENTARY MATERIAL

Automated Object Detection

Additional aspects of automated object detection are presented, which should investigate the generalization ability of our concept. First, the results regarding the detection of seeds are shown. Second, other results of the automated detection of segments are presented.

Seeds

In many cases, it is sufficient in high-throughput experiments to count objects, i.e., cells, instead of performing segmentation. This method is referred to as seed detection. Especially in terms of annotation effort, there is an advantage for users since only center points instead of contours have to be annotated. For less complex datasets, the seed detection analysis can be done using the Blob method [1] or the Otsu threshold method. In case of more complex problems, a DL-based method like BeadNet[2] may be helpful. Here, a DNN is utilized to predict centroids of objects.

In the following, we investigate the datasets DMA-Cell-Fluorescence, Insects2022[3], and LIVECell[4]. A quantitative comparison of the introduced method regarding the detection of seeds is depicted in Table 1. Likewise, qualitative results can be found in Figure 1. The DL approach is superior to the traditional image processing algorithm, especially in complex problems.

Segments

The method to obtain segments is already described in the manuscript. In addition, a comparison of the DL pipeline to the traditional Otsu method is considered. Quantitative results are displayed in Table 2 and qualitative results are shown in Figure 2. Analogous to the previous results, the DNNs are superior to Otsu. Consequently, we demonstrate the limitation of using a simple image processing module, such as Otsu.

Table 1. Comparison of Methods for Seed Detection. The performance of the Blob, Otsu and DL pipeline is evaluated using Dice Coefficient Q_{DSC} for the DMA-Cell-Fluorescence, Insects2022[3], and LIVECell[4] dataset. The superior method is highlighted in bold.

Method	DMA-Cell-Fluorescence	Insects2022	LIVECell
Blob	0.384	0.051	0.506
Otsu	0.413	0.089	0.158
DL	**0.850**	**0.775**	**0.841**

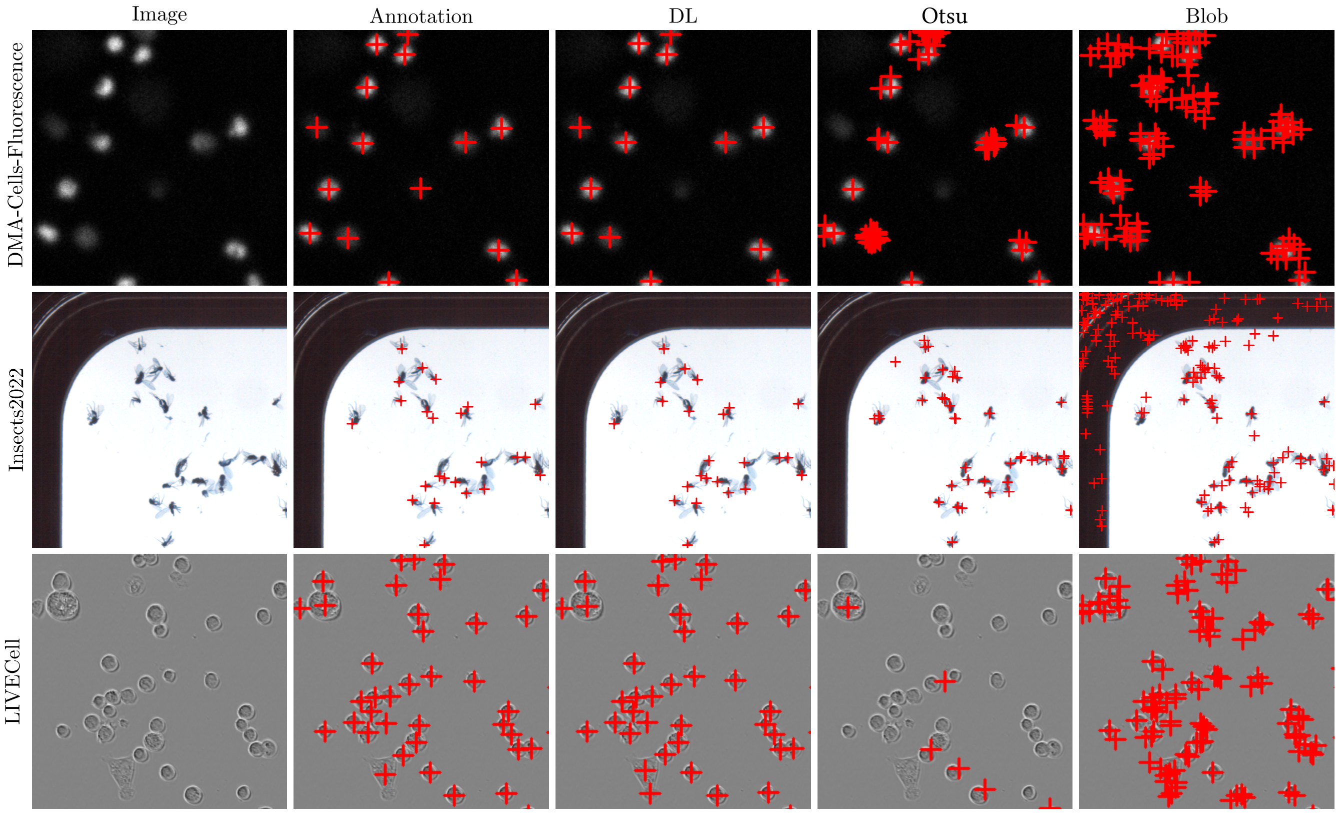

Figure 1. Predictions Using Seed Detection. For an example image of the three datasets (DMA-Cell-Fluorescence, Insects2022[3], and LIVECell[4]), the image including the annotation and the prediction of the different methods (DL, Otsu, Blob) of the seed detection analysis pipeline are shown. Red crosses mark the center points of the detected objects.

Table 2. Comparison of Methods for Segmentation. The performance of the Otsu and DL methods are quantified using Dice Coefficient Q_{DSC} in the case of semantic segmentation and using advanced Aggregated Jaccard Index $Q_{\mathrm{AJI+}}$ for instance segmentation, respectively, with the superior method highlighted in bold. Test images from the DMA-Cell-Fluorescence, Insects2022, DMA-Spheroid-BF, and DMA-Spheroid-Fluorescence datasets are analyzed.

Method	DMA-Cell-Fluorescence	Insects2022	DMA-Spheroid-BF	DMA-Spheroid-Fluorescence
Otsu	0.582	0.054	0.143	0.766
DL	**0.676**	**0.481**	**0.925**	**0.916**

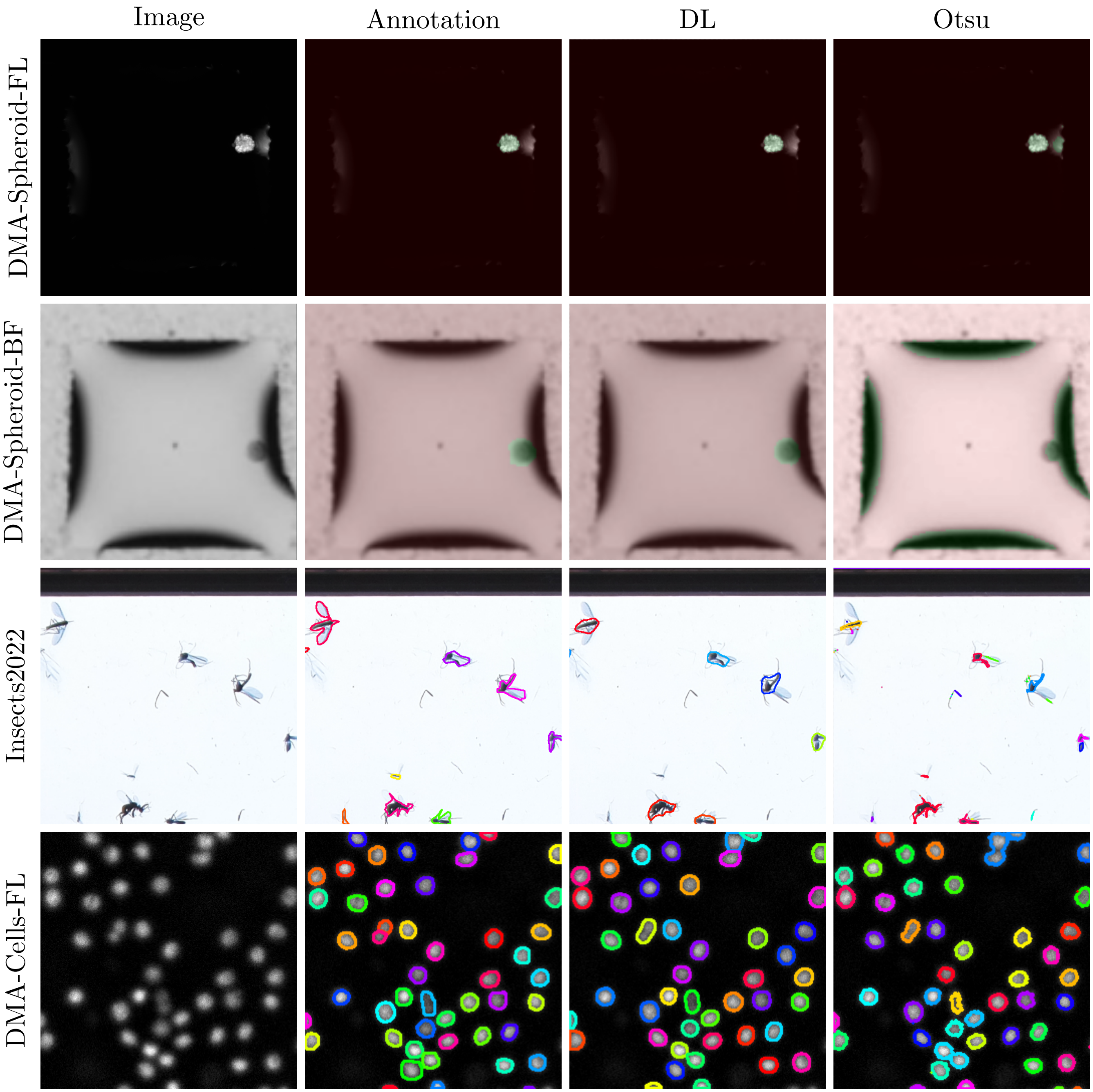

Figure 2. Prediction of Segments. For a single example of different datasets (DMA-Cell-Fluorescence, Insects2022, DMA-Spheroid-BF, and DMA-Spheroid-Fluorescence), a comparison between original image, annotation, and prediction of the DNN or the Otsu method is shown. In the case of semantic segmentation, segments are highlighted in light green, whereas the background is highlighted in light red. For instance segmentation, objects are color-coded by contours.

Colorimetric Analysis

Figure 3 displays another example of colorimetric analysis. Taking the true values known from a calibration measurement into account, the correlation between metric $\hat{y}_{i,j}$ and true value $\rho = 0.930$ is obtained. This finding aligns with the comparison of Figure 3 (a) and (b). Consequently, we show that our concept enables colorimetric analysis for another use case (different color spectrum) due to flexible transformation functions for quantification.

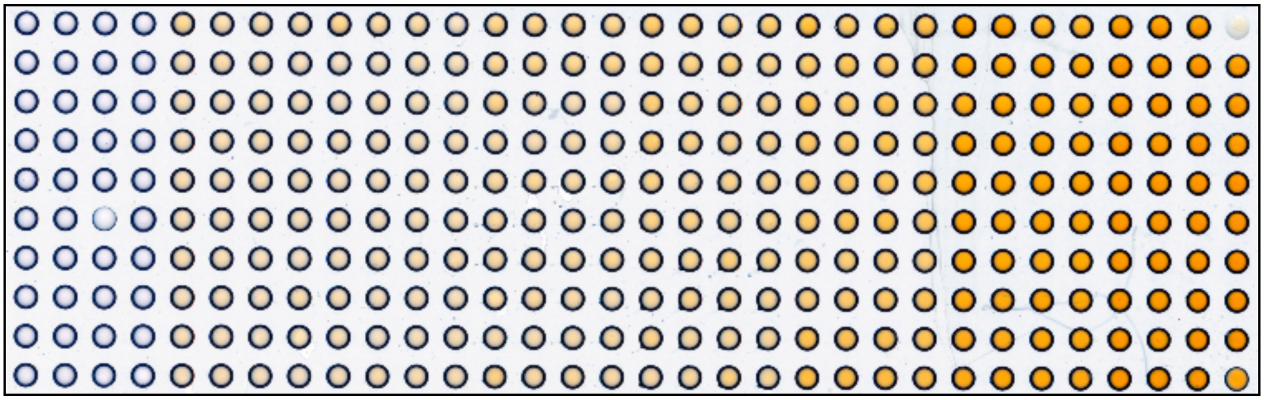

(a) Scanner Image.

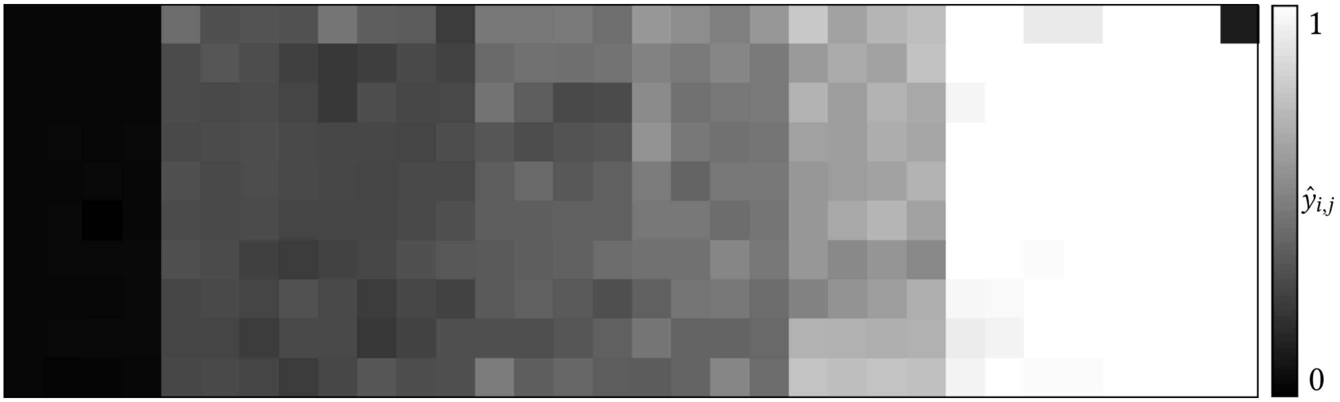

(b) Quantification.

Figure 3. Colorimetric Analysis. A scanner image (a) and the respective quantification $\hat{y}_{i,j}$ (b) are visualized.

References

1. Lindeberg, T. Feature detection with automatic scale selection. *Int. J. Comput. Vis.* **30**, 79–116 (1998).

2. Scherr, T. *et al.* BeadNet: Deep learning-based bead detection and counting in low-resolution microscopy images. *Bioinformatics* **36**, 4668–4670 (2020).

3. Wührl, L. *et al.* DiversityScanner: Robotic handling of small invertebrates with machine learning methods. *Mol. Ecol. Resour.* **22**, 1626–1638 (2022).

4. Edlund, C. *et al.* LIVECell—A large-scale dataset for label-free live cell segmentation. *Nat. Methods* **18**, 1038–1045 (2021).
